# Supplementary material for: Impact of Zinnia elegans Cultivation on the Control Efficacy and Distribution of Aphidius colemani Viereck (Hymenoptera: Braconidae) against Aphis gossypii Glover (Hemiptera: Aphididae) in Cucumber Greenhouses
Source: Insects. 2024 Oct 15;15(10):807. doi: 10.3390/insects15100807 (PMC11508801; doi:10.3390/insects15100807)
Supplement: Supplementary file 1 [file insects-15-00807-s001.zip › insects-3193553-supplementary/insects-3193553-supplementary-10.12/Supplementary figure.pptx]

## Slide 1
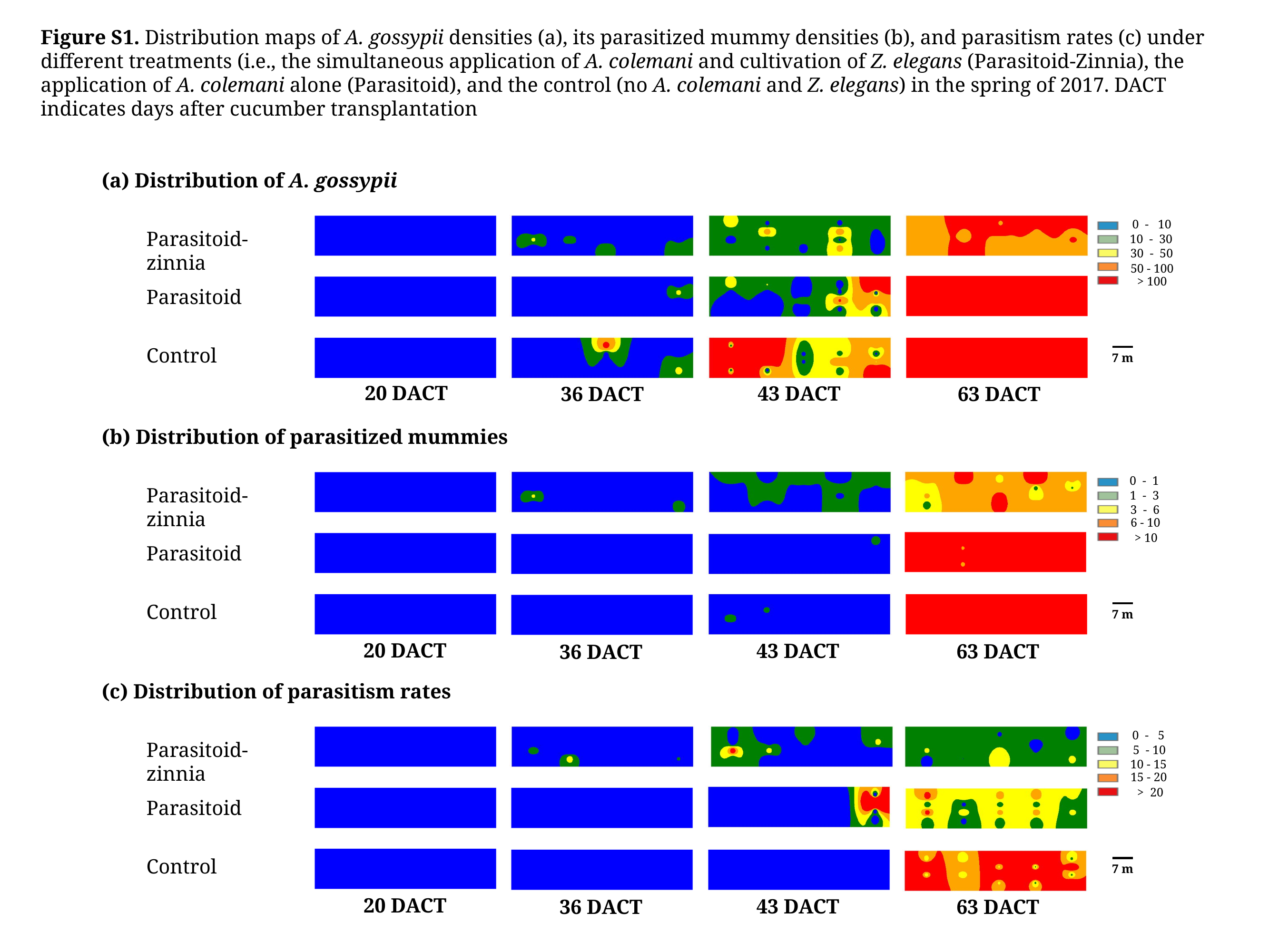

Figure S1. Distribution maps of A. gossypii densities (a), its parasitized mummy densities (b), and parasitism rates (c) under different treatments (i.e., the simultaneous application of A. colemani and cultivation of Z. elegans (Parasitoid-Zinnia), the application of A. colemani alone (Parasitoid), and the control (no A. colemani and Z. elegans) in the spring of 2017. DACT indicates days after cucumber transplantation
(a) Distribution of A. gossypii
0 - 10
Parasitoid-zinnia
 10 - 30
30 - 50
50 - 100
 > 100
Parasitoid
Control
7 m
20 DACT
43 DACT
 63 DACT
36 DACT
(b) Distribution of parasitized mummies
0 - 1
Parasitoid-zinnia
 1 - 3
3 - 6
6 - 10
 > 10
Parasitoid
Control
7 m
20 DACT
43 DACT
 63 DACT
36 DACT
(c) Distribution of parasitism rates
0 - 5
Parasitoid-zinnia
 5 - 10
10 - 15
15 - 20
 > 20
Parasitoid
Control
7 m
20 DACT
43 DACT
 63 DACT
36 DACT
