# Supplementary material for: Impact of Zinnia elegans Cultivation on the Control Efficacy and Distribution of Aphidius colemani Viereck (Hymenoptera: Braconidae) against Aphis gossypii Glover (Hemiptera: Aphididae) in Cucumber Greenhouses
Source: Insects. 2024 Oct 15;15(10):807. doi: 10.3390/insects15100807 (PMC11508801; doi:10.3390/insects15100807)
Supplement: Supplementary file 1 [file insects-15-00807-s001.zip › insects-3193553-supplementary/insects-3193553-supplementary-10.12/Supplementary table.pdf]

Table S1. Spatial distribution patterns and their related parameters of *A. gossypii*, its parasitized mummies and their parasitism rates using SADIE in three treatments during the spring trials of 2017.

Date (DACT)	Treatment								
	Natural enemies-zinnia			Natural enemies			Control		
	I_a	P_a	Pattern	I_a	P_a	Pattern	I_a	P_a	Pattern
Aphis gossypii									
20	N/A*	N/A	-	N/A	N/A	-	N/A	N/A	-
36	0.611	0.885	Random	1.411	0.039	Aggregated	0.746	0.846	Random
43	0.592	0.910	Random	1.374	0.103	Random	1.617	0.064	Random
63	0.785	0.718	Random	1.922	0.013	Aggregated	1.378	0.154	Random
Parasitized mummy									
20	N/A	N/A	-	N/A	N/A	-	N/A	N/A	-
36	0.775	0.808	Random	N/A	N/A	-	N/A	N/A	-
43	0.477	0.974	Uniform	0.789	0.744	Random	1.03	0.372	Random
63	0.739	0.744	Random	1.437	0.128	Random	0.695	0.795	Random
Parasitism rate									
20	N/A	N/A	-	N/A	N/A	-	N/A	N/A	-
36	0.812	0.615	Random	N/A	N/A	-	N/A	N/A	-
43	0.980	0.462	Random	1.186	0.321	Random	0.707	0.846	Random
63	0.709	0.744	Random	0.714	0.846	Random	0.663	0.846	Random

* N/A indicates that insect counts were insufficient to conduct aggregation analysis
